# Late Presentation of Transfusion-related Acute Lung Injury in the Emergency Department

**DOI:** 10.5811/cpcem.2018.11.40592

**Published:** 2019-01-07

**Authors:** David K. Peak, William T. Davis, Steven B. Walton

**Affiliations:** San Antonio Uniformed Services Health Education Consortium, Department of Emergency Medicine, San Antonio, Texas

## Abstract

Transfusion-related acute lung injury (TRALI) is a complication of blood product transfusion characterized by respiratory distress with bilateral lung infiltrates and non-cardiogenic pulmonary edema developing within six hours of transfusion. TRALI is believed to result from an immunological response to transfused blood products. TRALI is a clinical diagnosis that requires the exclusion of other etiologies of pulmonary edema and acute lung injury. Here we report a case of a female who presented to the emergency department in acute respiratory distress two days after receiving a transfusion of packed red blood cells for post-operative anemia following a hysterectomy.

## INTRODUCTION

Transfusion of blood products throughout calendar year 2015 was comprised of 11.3 million whole blood and red blood cells, 2.1 million apheresis platelets, and 3.6 million plasma transfusions. Complications arising from blood transfusions range from allergic reactions to hemolytic transfusion reactions, febrile transfusion reaction, transfusion-associated circulatory overload, and transfusion-related acute lung injury (TRALI).^1^ TRALI is a complex clinical syndrome that arises after transfusion of fresh frozen plasma, platelets, or packed red blood cells.^2^ TRALI occurs approximately once in every 5,000 transfusions.^3^ Although TRALI is usually a self-limiting process requiring minimal support, it can be fatal if not identified and treated early.^4^ Here we present a case of a 52-year-old female who developed TRALI after receiving two units of packed red blood cells for symptomatic post-operative anemia.

## CASE REPORT

A 52-year-old female with hypertension and no other past history of cardiopulmonary disease presented to the emergency department (ED) in acute respiratory distress. Two days prior to arrival, the patient underwent a total laparoscopic hysterectomy, which was complicated by a prolonged surgical course of six hours and an estimated blood loss of 1,500 milliliters. The patient suffered from symptomatic post-operative anemia and was given two units of packed red blood cells on post-operative day one. The patient experienced mild shortness of breath shortly after the transfusion but was cleared by pulmonology for discharge after maintaining normal vital signs during a trial of ambulation and lacking the appropriate clinical evidence to support a diagnosis of TRALI. The patient endorsed progressively worsening dyspnea at home, which prompted her to return to the ED less than 24 hours after being discharged.

Initial vital signs were notable for tachypnea to 30 breaths per minute, an oxygen saturation of 77% on room air, tachycardia to 107 beats per minute, blood pressure of 177/94 millimeters of mercury, and an oral temperature of 101.8° Fahrenheit. The patient was in moderate respiratory distress with suprasternal retractions, accessory muscle use, diffuse rales, anxiety, diaphoresis, and speaking in short phrases. Her electrocardiogram showed sinus tachycardia without evidence of acute ischemia or infarction.

**Image 1:**
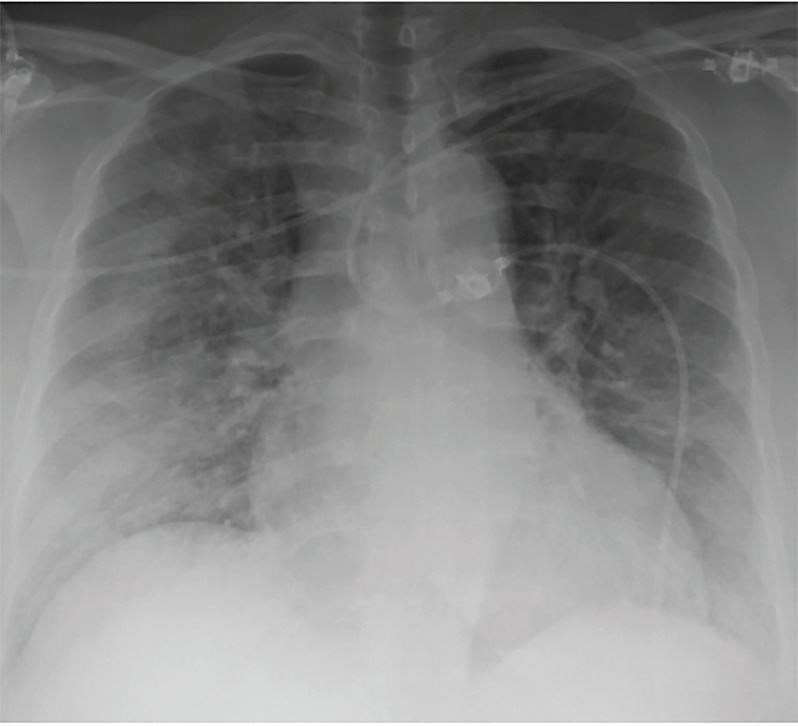
Anteroposterior chest radiograph in the emergency department showed bilateral pulmonary edema.

**Image 2:**
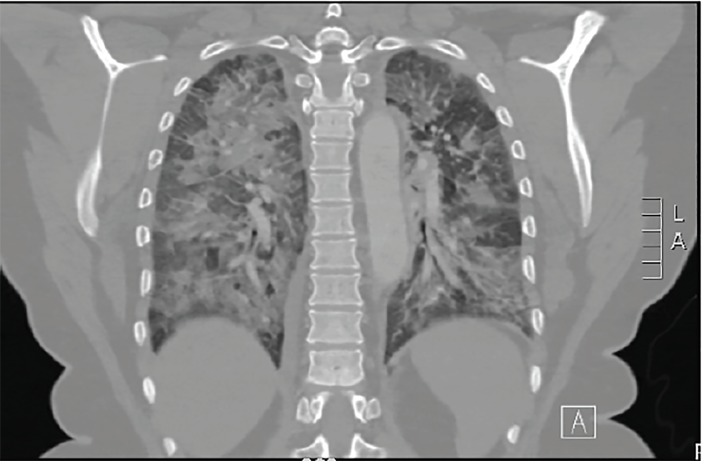
Coronal chest computed tomography demonstrated bilateral pulmonary edema.

Chest radiography (Image 1) and a computed tomography (CT)-pulmonary angiography (Image 2) revealed bilateral pulmonary edema, which was not present on prior imaging. No pulmonary embolism was seen. The patient remained hypoxic to 90% oxygen saturation despite receiving eight liters of oxygen by non-rebreather mask. The patient was given 40 milligrams (mg) intravenous (IV) furosemide, 0.4 mg sublingual nitroglycerin, and one gram IV acetaminophen. Her respiratory status remained unchanged with these interventions, so noninvasive ventilation was initiated with continuous positive airway pressure at five centimeters water.

The patient was admitted to the medical intensive care unit given her need for noninvasive ventilatory support. She underwent diuresis and weaning of her respiratory support. On hospital day three, the patient was discharged following complete resolution of her respiratory symptoms.

## DISCUSSION

TRALI is defined by the National Heart, Lung, and Blood Institute as acute lung injury within six hours of a blood product transfusion that cannot be explained by another cardiopulmonary process.^5^ TRALI occurs in approximately one in 5,000 patients receiving transfusions.^5^ The risk of TRALI varies depending on the blood product transfused.^6^ According to one study, one episode of TRALI occurred per 15,924 transfusions of fresh frozen plasma, 44,092 transfusions of red blood cells, 40,452 transfusions of whole blood platelet pools, and 47,000 transfusions of apheresis platelets.^7^ Patients receiving a greater number of units of blood products are at higher risk of developing TRALI.^2^ TRALI is now considered the leading cause of transfusion-related mortality in developed countries.^2^

There are two competing hypotheses as to the cause of TRALI. The “antigen-antibody hypothesis” postulates that alloantibodies in the donor blood product activate the recipient’s neutrophils, monocytes, or tissue macrophages. This immune system activation initiates an inflammatory cascade, damages the pulmonary endothelium, and increases the permeability of endothelial cells.^8^ The “two-event hypothesis” proposes that an initial event, such as surgery or underlying inflammation, increases the patient’s risk of TRALI. Then, lipids and cytokines from the transfused blood products act as the second event by activating neutrophils and causing pulmonary damage.^9^

**CPC-EM Capsule**

What do we already know about this clinical entity?

*Transfusion-related acute lung injury* (*TRALI) is characterized by respiratory distress and noncardiogenic pulmonary edema developing within six hours of receiving a blood transfusion*.

What makes this presentation of disease reportable?

*This patient presented to the emergency department (ED) more than 24 hours post-transfusion due to a progressive worsening of respiratory symptoms*.

What is the major learning point?

*TRALI is a possible etiology of respiratory distress even when the ED presentation is greater than six hours after a blood transfusion*.

How might this improve emergency medicine practice?

*A recent blood product transfusion is important history to consider for any patient presenting with respiratory distress*.

TRALI is a clinical diagnosis that can only be made after ruling out other causes of acute lung injury. Signs and symptoms associated with TRALI include hypoxemia, fulminant pulmonary edema, fever, tachycardia, and hypotension or hypertension.^2^ The clinical diagnosis of TRALI is made based on a history of recent blood product transfusion followed by signs and symptoms of acute lung injury. Echocardiography and B-type natriuretic peptide measurements may help in differentiating between hydrostatic causes of pulmonary edema such as transfusion-associated circulatory overload and the non-cardiogenic pulmonary edema seen in TRALI. Invasive techniques such as right heart catheterization and sampling of alveolar fluid protein may further help to classify the cause of acute pulmonary edema.^10^

Management of TRALI involves supportive care with oxygen supplementation and ventilatory assistance when appropriate.^11^ If mechanical ventilation is required, a low-tidal volume strategy is important to minimize additional ventilator-induced lung injury.^12^ There is currently not sufficient literature to support either the use of corticosteroids or statins. Conservative fluid practices are appropriate, as long as appropriate steps are made to avoid hypotension. Preventative strategies, such as conservative transfusion practices, avoiding high-plasma component donors, using fresh, un-stored red blood cells, and excluding plasma from female donors, have helped reduce the incidence of TRALI.^13^

## CONCLUSION

Our patient presented with respiratory distress from acute lung injury two days after receiving a blood transfusion. Although TRALI is defined as occurring within six hours of blood product transfusion, this case highlights the possibility of a delayed presentation of TRALI to the ED if the initial respiratory symptoms are not recognized. Emergency providers must keep TRALI on the differential for patients presenting with dyspnea after recently receiving any blood product transfusions.
